# Loss-of-function of *sox3* causes follicle development retardation and reduces fecundity in zebrafish

**DOI:** 10.1007/s13238-018-0603-y

**Published:** 2018-12-26

**Authors:** Qiang Hong, Cong Li, Ruhong Ying, Heming Lin, Jingqiu Li, Yu Zhao, Hanhua Cheng, Rongjia Zhou

**Affiliations:** Hubei Key Laboratory of Cell Homeostasis, College of Life Sciences, Wuhan University, Wuhan, 430072 China

**Keywords:** Sox3, follicle development, apoptosis, Cyp19a1a, zebrafish

## Abstract

Folliculogenesis is essential for production of female gametes in vertebrates. However, the molecular mechanisms underlying follicle development, particularly apoptosis regulation in ovary, remain elusive. Here, we generated *sox3* knockout zebrafish lines using CRISPR/Cas9. *sox3* knockout led to follicle development retardation and a reduced fecundity in females. Comparative analysis of transcriptome between *sox3*^−/−^ and wild-type ovaries revealed that Sox3 was involved in pathways of ovarian steroidogenesis and apoptosis. Knockout of *sox3* promoted follicle apoptosis and obvious apoptosis signals were detected in somatic cells of stages III and IV follicles of *sox3*^−/−^ ovaries. Moreover, Sox3 can bind to and activate the promoter of *cyp19a1a*. Up-regulation of Cyp19a1a expression promoted 17β-estradiol synthesis, which inhibited apoptosis in follicle development. Thus, Sox3 functions as a regulator of Cyp19a1a expression, via 17β-E2 linking apoptosis suppression, which is implicated in improving female fecundity.

**Electronic supplementary material:**

The online version of this article (10.1007/s13238-018-0603-y) contains supplementary material, which is available to authorized users.

## Introduction

In the vertebrate ovary, follicles are the functional units for oogenesis, which contain oocytes, granulosa cells and theca cells. The communication between oocytes and granulosa cells is essential for oocyte development (Eppig, 2001). Granulosa cells can provide some substances, including cholesterol, specific amino acids and nutrients for oocyte development (Su et al., 2009), while oocytes secrete some paracrine factors, such as BMP15 (bone morphogenetic protein 15) and GDF9 (growth differentiation factor 9) to regulate granulosa cells development (Su et al., 2004; Su et al., 2009). Hence, the bidirectional communication between oocytes and somatic cells is important for oogenesis. Studies have shown that autophagy of ovarian somatic cells is involved in regulation of follicular development by maintaining cell homeostasis (Yuan et al., 2015), while apoptosis of granulosa cells is pivotal for follicular development through atresia of many early follicles to ensure growth and maturation of some dominant follicles (Matsuda et al., 2012). However, the molecular mechanisms connecting apoptosis in ovary to follicle development remain largely unknown. Identification of key regulators of apoptosis and relevant pathways is important for understanding ovarian functions.

In follicle development, some factors and relevant pathways involved in both pro- and anti-apoptotic have been identified. FSH inhibited FoxO1-dependent apoptosis by coordinating the PKA-PI3K-AKT-FoxO1 axis and FoxO1-FoxO1 positive feedback in mouse granulosa cells (Shen et al., 2014). FGF-2 can inhibit apoptosis and promote follicle growth in culture *in vitro* in sheep (Santos et al., 2014). Up-regulated apoptosis was associated with a decrease in number of vitellogenic follicles when the physiochemical waters parameters were unfavorable in fish (Thome et al., 2012). BCL2 family members, which included pro-apoptotic (e.g., *Bax* and *Bok*) and anti-apoptotic (e.g., *Bcl2* and *Mcl1*) genes, were another kind of apoptosis regulators during follicle development (Perez et al., 1999; Hutt, 2015). *Bcl2* knockout led to aberrant follicle development in adult mice (Ratts et al., 1995), while overexpression of *Bcl2* resulted in decreased follicle apoptosis (Hsu et al., 1996). However, *Bcl2* knockout did not alter neonatal ovarian histology (Jones and Pepling, 2013). In addition, estradiol and microRNA can inhibit granulosa cell apoptosis and promote follicular development (Liu et al., 2014b; Qiu et al., 2014). Despite these progresses, the molecular mechanisms of ovarian apoptosis and its influence on follicle development remain elusive.

Zebrafish (*Danio rerio*), as an excellent model organism, has two distinct sexes in adults, but they are juvenile hermaphrodites (Takahashi, 1977). All the gonads of early-stage (3 weeks ago) are undifferentiated gonads, called “bipotential juvenile ovaries” (Takahashi, 1977; Wang et al., 2007b). From 20 days post fertilization (dpf), some of the bipotential ovaries continue to become ovaries, the others undergo an apoptosis process and finally develop into testes. About 40 dpf, their sexes can be distinguished according to gonadal histology (Uchida et al., 2002; Orban et al., 2009). Molecular mechanisms of sex determination in zebrafish remain elusive. Domesticated zebrafish strains lack sex-linked loci, although natural strains have WZ/ZZ sex chromosomes (Wilson et al., 2014). A number of genes involved in gonadal differentiation have been identified in zebrafish, which include female sex-biased genes: *mettl3* (methyltransferase like 3) (Xia et al., 2018), *cyp19a1a* (cytochrome P450, family 19, subfamily A, polypeptide 1a) (Lau et al., 2016; Yin et al., 2017), *nr0b1* (nuclear receptor subfamily 0 group B member 1) (Chen et al., 2016), *foxl2a* (forkhead box L2a), *foxl2b* (forkhead box L2b) (Yang et al., 2017), *bmp15* (bone morphogenic protein 15) (Dranow et al., 2016) and *fgf24* (fibroblast growth factor 24) (Leerberg et al., 2017), and male sex-biased genes: *dmrt1* (doublesex and mab-3 related transcription factor 1) (Guo et al., 2005; Lin et al., 2017; Webster et al., 2017), *amh* (anti-Mullerian hormone) (Lin et al., 2017), *sox9a* (sex-determining region Y-box 9a) (Sun et al., 2013) and *ar* (androgen receptor) (Crowder et al., 2018). As lack of morphological sex chromosome in zebrafish, molecular mechanisms of sex determination and differentiation are probably multigenic (Liew et al., 2012) and key genes remain to be identified.

*Sox3* (sex-determining region Y-box 3), belonged to the SOX family, is an ancestral precursor of *Sry* (Foster and Graves, 1994), which is a key male sex-determining gene in mammals (Sinclair et al., 1990; Koopman et al., 1991). In transgenic mice, overexpression of *Sox3* led to a complete XX male sex reversal phenotype (Sutton et al., 2011), while loss-of-function mutations showed that it was not required for sex determination, but important for oocyte development, testis differentiation and gametogenesis (Weiss et al., 2003). Nevertheless, genomic rearrangements, de novo duplication or interchromosomal insertional translocation at xq26.3 regulatory region of SOX3 caused XX male sex reversal in humans (Sutton et al., 2011; Moalem et al., 2012; Haines et al., 2015). *Sox3* was also required for formation of the hypothalamo-pituitary axis in mice (Rizzoti et al., 2004), the neurogenesis and neural tube in chicken (Bylund et al., 2003) and zebrafish (Dee et al., 2008; Gou et al., 2018a; Gou et al., 2018b). In addition, in medaka (*Oryzias dancena*), *sox3*^*Y*^ was a male-determining factor (Takehana et al., 2014). However, *sox3* had more important role in oogenesis than in spermatogenesis in grouper (*Epinephelus coioides*) (Yao et al., 2007) and Japanese eel (*Anguilla japonica*) (Jeng et al., 2018). Thus, functions of *sox3* are complex and multiple across vertebrates.

In the present study, we first generated *sox3* knockout zebrafish lines using CRISPR/Cas9 and found that *sox3* knockout led to follicle development retardation and a reduced fecundity in females. Transcriptome analysis revealed that apoptosis signaling pathway was up-regulated and ovarian steroidogenesis was down-regulated in *sox3*^−/−^ ovaries in comparison with wild type ovaries. Knockout of *sox3* caused follicle apoptosis. Furthermore, we demonstrated that Sox3 can promote 17β-E2 synthesis by binding to and activating the *cyp19a1a* promoter, which led to apoptosis decrease in follicle development. Hence, we revealed Sox3 as a regulator of Cyp19a1a expression, via 17β-E2 linking apoptosis suppression in ovary development, which is implicated in improving female fecundity.

## Results

### Generation of *sox3* mutant lines using CRISPR/Cas9

**Figure 1:**
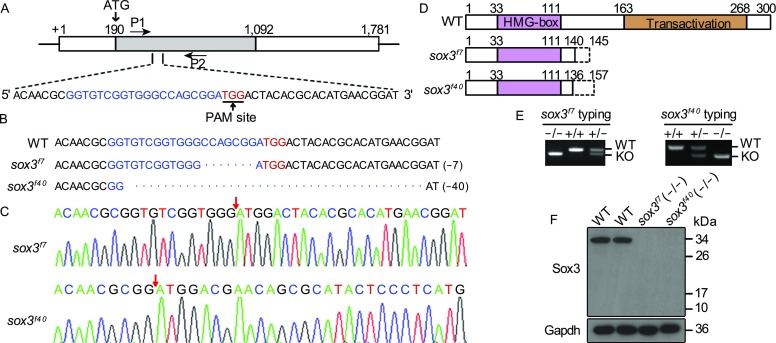
**Generation of** ***sox3*** **mutant strains of zebrafish using CRISPR/Cas9 system**. (A) Schematic representation of gRNA targeting the *sox3* locus. The translation start codon ATG was indicated by an arrow. The gRNA (blue) was designed to target open reading frame (ORF) (gray). The PAM (protospacer adjacent motif) site was underlined in red. P1 and P2 were primers for genotyping. (B) Nucleotide sequence alignments of the two mutant sites (*sox3*^*f7*^ and *sox3*^*f40*^) with WT sequence. The deleted sequences were indicated in dotted lines. One had a 7-bp deletion, the other had a 40-bp deletion. (C) DNA sequencing of *sox3* mutant alleles of both *sox3*^*f7*^ and *sox3*^*f40*^ strains. The red arrows indicated the deleted positions. (D) Schematic diagram of protein coding regions of wide type Sox3 and two predicted truncated mutants. The conserved domains (HMG-box and transactivation) were indicated in boxes (solid lines). Frameshift sequences were showed in boxes with dash lines. The numbers refer to the amino acid positions. (E) PCR analysis to determine genotypes of offsprings from heterozygote intercrosses in two KO strains. The KO band (287 bp of *sox3*^*f7*^ and 254 bp of *sox3*^*f40*^) can be distinguished from WT band (294 bp). Primer sequences and PCR conditions were listed in Table S1. (F) Western blot analysis of Sox3 expression. Ovary extracts from adult zebrafish were subjected to Western blots to detect Sox3 expression. The band of Sox3 protein was present in 33 kDa in WT zebrafish. Gapdh was used as an internal control

To explore the function of *sox3* in ovary development in zebrafish, *sox3* knockout zebrafish lines were first generated using CRISPR/Cas9 technology. We used a CRISPR design web tool (http://crispr.mit.edu/) to design gRNA targeted the 5′ upstream of coding region (Fig. 1A). Two independent *sox3* mutant lines were established: one had a 7-bp deletion (*sox3*^*f7*^) and the other had a 40-bp deletion (*sox3*^*f40*^) (Fig. 1B and 1C). Both induced frame-shift mutations (Fig. 1D). Sox3 has a DNA-binding domain and a transactivation domain. To determine loss of function in both *sox3*^*f7*^ and *sox3*^*f40*^, deletion mapping and Gal4 dual-luciferase reporter assay system were performed, which revealed that the transactivation domain is essential for transcriptional activation of Sox3 (Figs. S1A and 1B). To characterize both *sox3*^*f7*^ and *sox3*^*f40*^ zebrafish at the molecular level, quantitative real-time PCR was performed, which revealed that *sox3* was significantly reduced in *sox3* knockout ovaries compared to wild type ovaries (Fig. S2), suggesting transcript destabilization and degradation of Sox3^f7^ and Sox3^f40^ in the mutants by nonsense-mediated mRNA decay (NMD) due to premature termination codons (Baker and Parker, 2004; Popp and Maquat, 2016). Homozygotes (*sox3*^−/−^) were produced by incross of the *sox3*^+/−^ zebrafish, which can be identified by PCR (Fig. 1E). Western blot analysis showed complete absence of Sox3 protein in the both mutant lines (Fig. 1F). Thus, both mutant lines had lost the function of Sox3. In order to determine the off-target effects, we aligned the target sequence to the zebrafish genome to search for all the potential off-target sites using the on-line tool (http://crispr.mit.edu). The results indicated that no off-target mutagenesis was detected in the two mutant lines (Fig. S3A–C). These data suggested that Sox3 was disrupted with no protein expression in the two knockout lines.

### Sox3 is required for follicle development and fecundity in zebrafish

**Figure 2:**
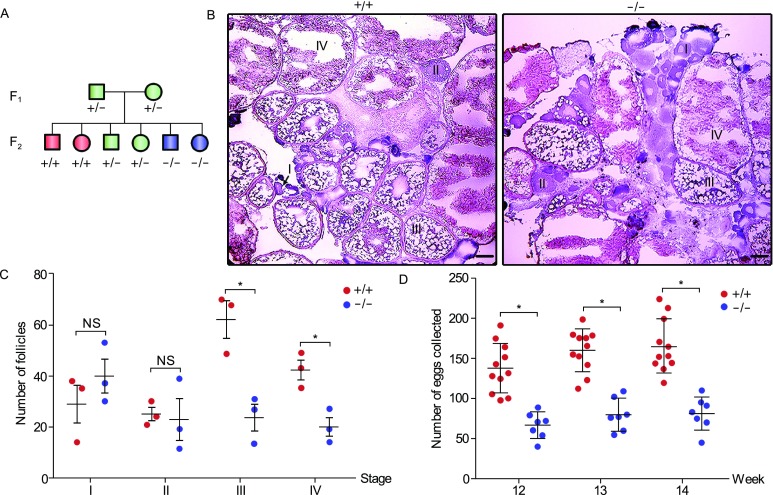
**Knockout of** ***sox3*** **leads to follicle development retardation and a reduced fecundity in female zebrafish**. (A) Schematic diagram of heterozygote incrosses. All data are from the progeny derived from heterozygote incrosses. WT, +/+; heterozygotes, +/−; homozygotes, −/−. (B) Histological analysis of adult ovaries of *sox3*^+/+^ and *sox3*^−/−^ by H.E. staining. I, primary growth stage; II, cortical alveolus stage; III, early-vitellogenic stage; IV, late-vitellogenic stage. Scale bar, 50 μm. (C) Statistic analysis of different stages of follicles from 10 sections for each ovary of *sox3*^+/+^ and *sox3*^−/−^ (*n* = 3 fish per genotype). Data represent means ± SEM. T-test was performed. *P < 0.05; **P < 0.01. NS, not significant. (D) Impaired fecundity in *sox3*^−/−^. Statistical analysis of the numbers of eggs produced by *sox3*^−/−^ incrosses or *sox3*^+/+^ incrosses at 12-, 13- and 14-week. Data represent means ± SEM. T-test was performed. *P < 0.05

To investigate roles of *sox3* in follicle development, both *sox3*^+/+^ and *sox3*^−/−^ from *sox3*^+/−^ incross were used for histological analysis of different stages of follicles. Statistical analysis showed less follicles of early-vitellogenic stage (III) and late-vitellogenic stage (IV) in *sox3*^−/−^ ovaries than those in wild-type ovaries. The follicle number of primary growth stage (I) and cortical alveolus stage (II) had no significant difference between *sox3*^+/+^ and *sox3*^−/−^ ovaries (Fig. 2A–C). These results indicated that follicle development was retarded in the *sox3*^−/−^ ovaries compared to wild type ovaries. Furthermore, the average number of eggs produced by females from *sox3*^−/−^ incrossed was significantly less than that from *sox3*^+/+^ incrossed at 12-, 13- and 14-week (Fig. 2D). Taken together, these results suggested that *sox3* knockout leads to follicle development retardation and a reduced fecundity in female zebrafish.

### Sox3 deletion influences pathways of ovarian steroidogenesis and apoptosis

**Figure 3:**
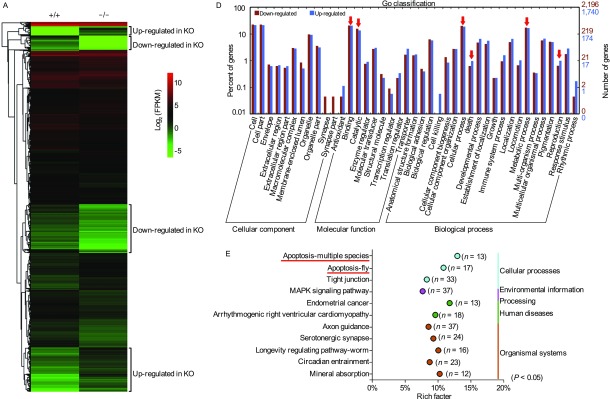

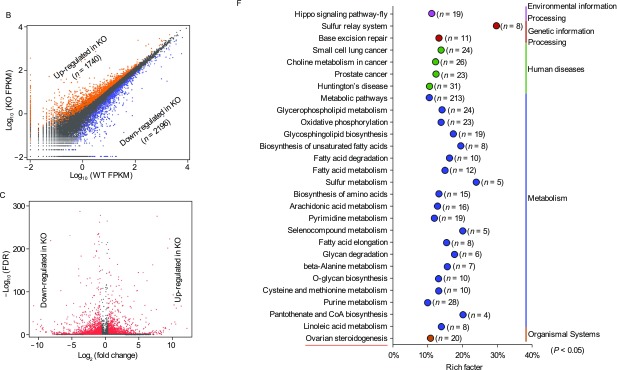
**Transcriptome analysis of adult ovaries from** ***sox3***^**+/+**^ **and** ***sox3***^**−/−**^. (A) Hierarchical clustering indicated the differentially expressed genes (DEGs) and their expression levels in *sox3*^+/+^ and *sox3*^−/−^ ovaries (fold change > 1.4 & FDR < 0.05). Colors indicated relative expression compared to the mean value. The scale bar indicated Log_2_(FPKM). FPKM, fragments per kilo bases per million fragments. (B) Scatter plot chart indicated the DEGs between *sox3*^+/+^ and *sox3*^−/−^ ovaries. Yellow and blue dots represented up-regulated (1,740, 8.44%) and down-regulated (2,196, 10.66%) genes in *sox3*^−/−^ ovaries, while the black dots represented equally expressed genes between *sox3*^+/+^ and *sox3*^−/−^ ovaries. (C) Volcano chart indicated the DEGs between *sox3*^+/+^ and *sox3*^−/−^ ovaries. Red dots represented up-regulated or down-regulated genes, while the black dots represented equally expressed genes between *sox3*^+/+^ and *sox3*^−/−^ ovaries. FDR, false discovery rate. (D) Gene Ontology (GO) analysis of DEGs between *sox3*^+/+^ and *sox3*^−/−^ ovaries. X-axis indicated the catalogs of cellular component, molecular function and biological process. Y-axis on the left indicated the number of genes in a category as a percent of the total number of DEGs and the actual numbers of DEGs are shown on the right. (E) KEGG analysis of up-regulated DEGs. The numbers represented the actual number of DEGs that were classified in a particular pathway. X-axis indicated the percentage of the DEGs in the pathway. (F) KEGG analysis of down-regulated DEGs. The numbers represented the actual number of DEGs that were classified in a particular pathway. X-axis indicated the percentage of the DEGs in the pathway

To examine possible pathways of Sox3 regulation in ovary development, we performed transcriptome sequencing and analysis of *sox3*^+/+^ and *sox3*^−/−^ ovaries (Fig. S4A–C and Tables S2–4). Over 3930 differentially expressed genes (DEGs) were detected, based on both fold change (FC) > 1.4 and false discovery rate (FDR) < 0.05, in the *sox3*^−/−^ ovaries in comparison with wild type ovaries, of them 8.44% (1740) were up-regulated and 10.66% (2,196) were down-regulated (Fig. 3A–C). Gene Ontology (GO) analysis of DEGs between *sox3*^+/+^ and *sox3*^−/−^ ovaries showed that the DEGs were mainly enriched in the binding and catalytic activity in molecular function, and cellular process, death, metabolic process and reproduction in biological process (Fig. 3D). Kyoto Encyclopedia of Genes and Genomes (KEGG) analysis showed that the apoptosis signaling pathway was significantly up-regulated and ovarian steroidogenesis was significantly down-regulated in *sox3*^−/−^ ovaries in comparison with wild type ovaries (Fig. 3E and 3F). These data suggested that Sox3 probably regulated ovary development through pathways of ovarian steroidogenesis and apoptosis.

### Disrupted pathway of 17β-E2 synthesis and up-regulated apoptosis in the *sox3*^−/−^ ovaries

**Figure 4:**
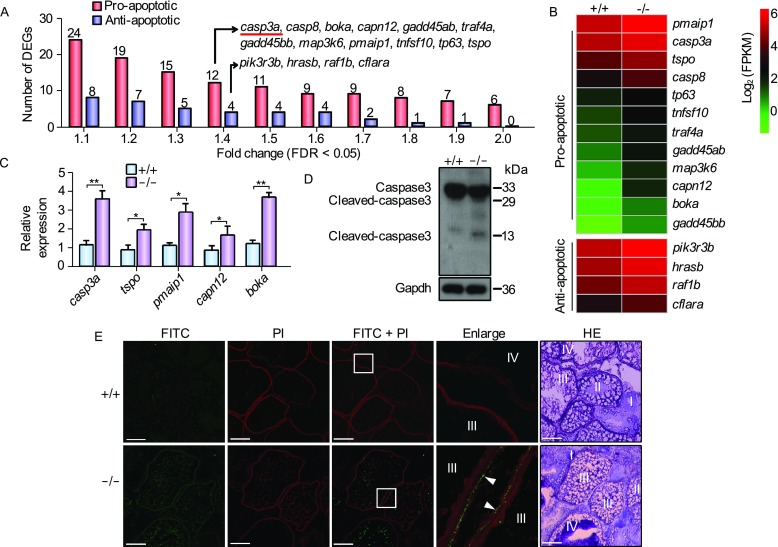
**Up-regulated apoptosis in the** ***sox3***^**−/−**^ **ovaries in comparison with wild type ovaries**. (A) Statistical analysis of the apoptosis-related DEGs (FDR < 0.05) in both *sox3*^+/+^ and *sox3*^−/−^ based on KEGG analysis. Red bars indicated pro-apoptotic genes and blue bars represented anti-apoptotic genes. The genes with fold change of 1.4 were listed. (B) Hierarchical clustering indicated the expression levels of apoptosis-related DEGs between *sox3*^+/+^ and *sox3*^−/−^ ovaries. (C) Quantitative real-time PCR analysis of selected genes to validate the DEGs from RNA-seq data. The transcript levels were related to *β-actin* expression. Relative level, 2^−ΔΔCt^. T-test was performed. *P < 0.05; **P < 0.01. (D) Western blot analysis showed that cleaved-caspase3 were up-regulated in the *sox3*^−/−^ ovaries in comparison with the wild type ovaries. Gapdh was used as an internal control. (E) TUNEL analysis. In follicles of stages III and IV, obvious signals (FITC-labeled, green) were observed in somatic cells (theca cells and granulosa cells) (white arrowheads) of *sox3*^−/−^ ovaries. The nuclei were stained by PI (red). The enlarged images originated from the white squares. The ovaries sections were stained by H.E. and showed on the right. Scale bar: 100 µm

To further explore molecular mechanism of Sox3-regulated apoptosis in ovary, transcriptome and quantitative real-time PCR analysis were then performed. Pro-apoptotic DEGs were significantly up-regulated in *sox3* knockout ovaries compared to wild type ovaries, such as *casp3a* (caspase 3, apoptosis-related cysteine peptidase a), *pmaip1* (phorbol-12-myristate-13-acetate-induced protein 1) and *tspo* (translocator protein), while a few of anti-apoptotic DEGs were also up-regulated in *sox3* knockout ovaries compared to wild type ovaries (Fig. 4A–C). Western blot analysis showed that cleaved-caspase3 was up-regulated in *sox3* knockout ovaries in comparison with wild type (Fig. 4D). TUNEL analysis further confirmed the up-regulated apoptosis in the *sox3*^−/−^ ovaries. In particular, in follicles of stages III and IV, obvious FITC-labeled signals were detected in somatic cells (theca cells and granulosa cells) of *sox3*^−/−^ ovaries (Fig. 4E). These results indicated that knockout of *sox3* promoted follicle apoptosis in zebrafish ovary.

**Figure 5:**
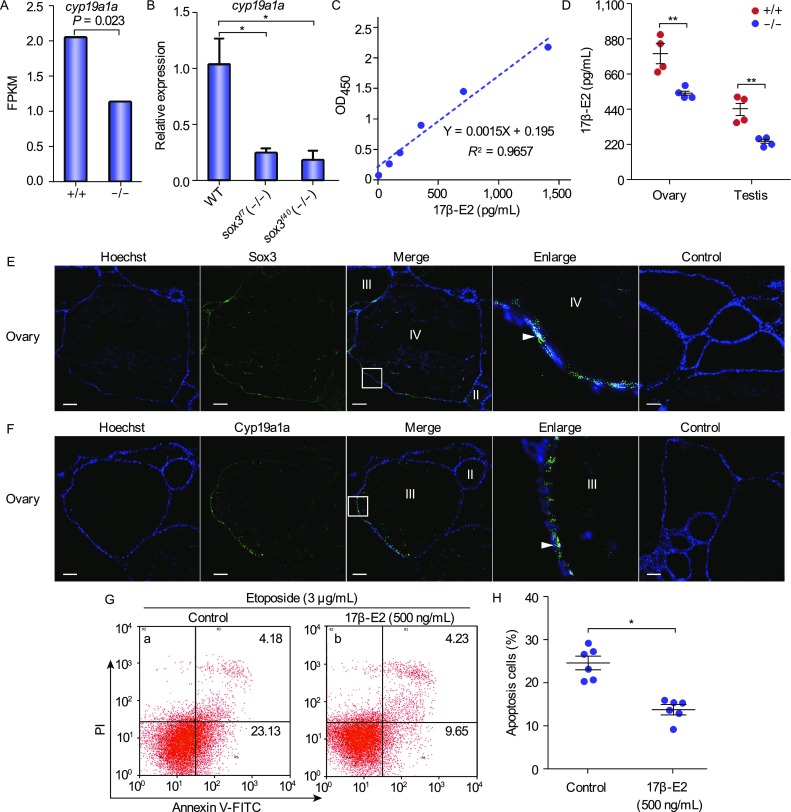
**Disrupted pathway of 17β-E2 synthesis and up-regulated apoptosis in the** ***sox3***^**−/−**^ **ovaries**. (A) The expression levels of *cyp19a1a* in both *sox3*^+/+^ and *sox3*^−/−^ ovaries based on RNA-seq data. (B) Quantitative real-time PCR analysis of *cyp19a1a* expression levels in both *sox3*^+/+^ and *sox3*^−/−^ ovaries. The transcript levels were related to *β-actin* expression. Relative level, 2^−ΔΔCt^. T-test was performed. *P < 0.05. (C) The standard curve of 17β-E2 concentration at OD_450_. (D) Down-regulated 17β-E2 in *sox3*^−/−^ gonads in comparison with the wild type gonads. Data represented means ± SEM. T-test was performed. *P < 0.05; **P < 0.01. (E) Immunofluorescence analysis of Sox3 protein in adult ovary. Anti-Sox3 and FITC-conjugated goat anti-rabbit IgG (H + L) antibodies were used to detect Sox3 (green). The nuclei were stained by Hoechst (blue). Preimmune serum was used as a negative control. The white square area in the inset was enlarged and showed on the right. Sox3 positive signals were observed in somatic cells (theca cells and granulosa cells) (white arrowheads) in ovary. Scale bar, 50 μm. (F) Immunofluorescence analysis of Cyp19a1a protein in adult ovary. Anti-Cyp19a1a and FITC-conjugated goat anti-rabbit IgG (H + L) antibodies were used to detect Cyp19a1a (green). The nuclei were stained by Hoechst (blue). Preimmune serum was used as a negative control. The white square area in the inset was enlarged and showed on the right. Cyp19a1a positive signals were observed in somatic cells (theca cells and granulosa cells) (white arrowheads) in ovary. Scale bar, 50 μm. (G) Assessment of apoptosis using Annexin V-FITC/PI and flow cytometry. CHO cells were treated with 17β-E2 (500 ng/mL) or DMEM (control) for 36 h, and then treated with etoposide (3 μg/mL) for 12 h and assayed by flow cytometry. Viable cells exhibited Annexin V-/PI- (symbol 3 in the plot); early apoptotic cells exhibited Annexin V+/PI-(symbol 4 in the plot); late apoptotic cells exhibited Annexin V+/PI+ (symbol 1 in the plot); necrotic cells and some late apoptotic cells exhibited Annexin V-/PI+ (symbol 2 in the plot). (H) Percentages of both early and late apoptotic cells based on the apoptosis assessment by flow cytometry and Annexin V/PI in (G). T-test was performed. *P < 0.05

To investigate how depletion of *sox3* influences ovary apoptosis, we analyzed pathways of Cyp19a1a regulation. Because Cyp19a1a can promote 17β-E2 synthesis (Miller, 2017), we examined possible association of *cyp19a1a* expression with 17β-E2 production in ovary. Synthesis level of 17β-E2 were indeed decreased in ovaries, while expression level of *cyp19a1a* in ovaries were significantly down-regulated in two knockout lines in comparison with wild type (Fig. 5A–D). Immunofluorescence analysis further showed that both Sox3 and Cyp19a1a proteins had a similar expression pattern in somatic cells (theca cells and granulosa cells) in ovary (Fig. 5E and 5F). To further confirm regulation relationship between 17β-E2 and apoptosis, we treated CHO cells with 17β-E2 and then added etoposide to test apoptosis level. Addition of 17β-E2 significantly inhibited apoptosis level in etoposide-induced CHO cells (Fig. 5G and 5H). These results suggested that Sox3 up-regulated *cyp19a1a* expression, which promoted 17β-E2 synthesis, thus inhibited apoptosis in ovary development.

### Sox3 binds to and activates *cyp19a1a* promoter in zebrafish

**Figure 6:**
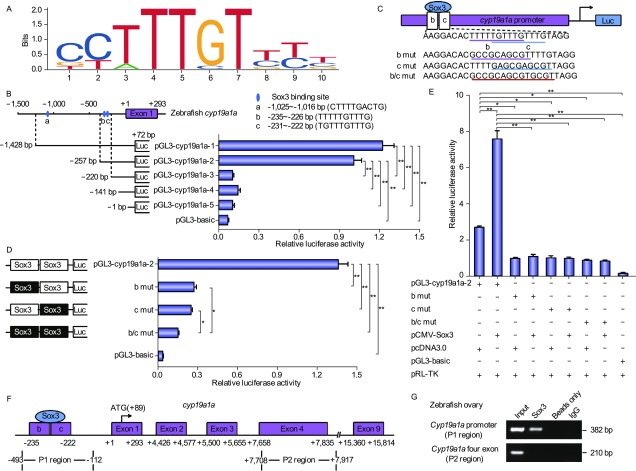
**Activity analysis of** ***cyp19a1a*** **promoter**. (A) Sequence logo of Sox3 binding site based on JASPAR database. (B) Luciferase assay showed the activities of a series of deleted constructs in HEK293T cells, which corresponded to three *sox3* binding sites (a, b and c). Left panel showed each deleted mutant linked with the luciferase gene in the pGL3-basic vector. Right panel indicated the relative activities of these deleted constructs, as determined by luciferase assays. One-way ANOVA was performed. *P < 0.05; **P < 0.01. (C) The DNA sequences of mutants (b, c and b/c) and wild type *cyp19a1a*. (D) Point mutation analysis of the core promoter using luciferase assays. The 329 bp pGL3-cyp19a1a-2 construct was used as a basic construct for the point mutation analysis. Luciferase assays were used to determine the relative activities. The intact binding sites of the Sox3 were indicated by open boxes, respectively. The filled boxes showed corresponding mutation. The pGL3-basic vector was used as a negative control. The data were based on the luciferase activity of pGL3-cyp19a1a-2. The means ± SD are from 3 independent experiments. One-way ANOVA was performed. *P < 0.05; **P < 0.01. (E) Overexpression of Sox3 up-regulated *cyp19a1a* promoter activity. HEK293T cells were transfected with 0.32 μg pGL3-cyp19a1a-2 or its several mutants (b, c and b/c) and 0.08 μg Sox3 expression plasmid (pCMV-Sox3), together with 10 ng pRL-TK, as indicated. One-way ANOVA was performed. *P < 0.05; **P < 0.01. (F) Schematic diagram of primer relative positions in the ChIP assay. (G) Chromatin immunoprecipitation analysis showed that Sox3 can bind to the *cyp19a1a* promoter *in vivo*. Sonicated chromatin from zebrafish ovaries were immunoprecipitated with anti-Sox3, no antibody (beads only) and preimmune IgG (control). A 382 bp fragment corresponding to the −493 to −112 region of the *cyp19a1a* promoter was amplified using the immunoprecipitated DNA as a template. Exon 4 of *cyp19a1a* was used as a negative control

To examine the role of Sox3 in regulating *cyp19a1a* expression in zebrafish, a series of deletions of the *cyp19a1a* promoter were used to identify possible binding sites of Sox3. Three Sox3 binding sites (a, b and c) were detected with a consensus motif according to JASPAR database (Fig. 6A and 6B). To identify the exact binding sites, luciferase activities in a series of deletions were examined. The results showed that the sequence from −257 to −220 bp in the 5′ flanking region was key for *cyp19a1a* transcriptional activity, which harbored two Sox3 binding sites (b and c) (Fig. 6A and 6B). To identify the roles of these sites, site-directed mutants were constructed using wild-type pGL3-cyp19a1a-2 plasmid as the template. Compared with the wild-type pGL3-cyp19a1a-2 construct, a single mutant showed an obvious decrease in promoter activity, and the activity was obviously deprived in double mutants (Fig. 6C and 6D). Furthermore, Sox3 transfection can up-regulate the pGL3-cyp19a1a-2-driven luciferase activity, whereas Sox3 had no effect on the promoter activities of the single or double sites mutation-driven luciferase activity, suggesting that Sox3 can bind to both sites in the *cyp19a1a* promoter and activate the promoter activity (Fig. 6E). Chromatin immunoprecipitation analysis was further performed to investigate whether Sox3 binds to the *cyp19a1a* promoter *in vivo*. A 382 bp DNA region from anti-Sox3 antibody precipitates was amplified in ovary, which was confirmed by sequencing. Another distinct genomic region (exon 4) was used as a control to exclude the possibility of nonspecific binding to the *cyp19a1a* promoter region. In the control, no band from the anti-Sox3 antibody precipitates was observed (Fig. 6F and 6G). These results suggested that Sox3 can bind to both sites (b and c) in the *cyp19a1a* promoter and activate the promoter activity in the zebrafish ovary.

## Discussion

Folliculogenesis is an important event for production of functional eggs in vertebrates, which could rely on the balance between pro-apoptosis and anti-apoptosis. However, the regulatory mechanisms underlying folliculogenesis via apoptosis remain elusive. In the present study, we provided a molecular mechanism of apoptosis-regulated follicle development in zebrafish. We revealed the Sox3 as a regulator of Cyp19a1a expression, which can inhibit ovarian apoptosis via 17β-E2 to ensure dominant follicle development. Sox3 knockout can disrupt the regulatory pathway, lead to apoptosis of granulosa cells and theca cells in ovary, thus retardation of follicle development and a reduced fecundity in females. These findings have potential implications in improving female fecundity through the apoptosis suppression pathway.

We have demonstrated that *sox3* is required for follicle development and fecundity in zebrafish. Excess follicular atresia and severely reduced fertility in the *sox3* KO mice were observed (Weiss et al., 2003; Rizzoti et al., 2004), which might be caused by apoptosis. Here, we determined the molecular connection between apoptosis and follicle development retardation by the Sox3-Cyp19a1a. In addition to gonad development, Sox3 was associated with X-linked mental retardation, growth hormone deficiency and X-linked panhypopituitarism in humans (Laumonnier et al., 2002; Bauters et al., 2014; Jelsig et al., 2018), and was required for the functions of pituitary and formation of midline structures in central nervous system in mice (Rizzoti et al., 2004). Knockdown of Sox3 resulted in a reduction in the size of the CNS, ears and eyes and inhibition of neurogenesis (Dee et al., 2008), while both Sox3 and Sox2 regulated otic/epibranchial placode induction and inner ear development in zebrafish (Gou et al., 2018a; Gou et al., 2018b). Besides, a decreased viability phenotype in the *sox3* knockout zebrafish was observed in our study, which is similar to that in the *sox3* knockout mice (~43% death before weaning) (Rizzoti et al., 2004). Thus, Sox3 has dual functions in development of both gonad and brain.

Sox3, as a member of SOXB1 family, had a DNA-binding domain and a transactivation domain. The transactivation domain is essential for transcriptional activation of *sox3*, while the DNA-binding domain is for targeting of its downstream genes. Both *sox3*^*f7*^ and *sox3*^*f40*^ mutants had loss of function of Sox3 in zebrafish, as deletion of transactivation domain. Deletions of either of these two domains led to the loss-of-function of *sox3* for oncogenic transformation in chicken embryo fibroblasts (Xia et al., 2000). Similarly, the full-length of *sox3* could rescue the morphological defect caused by the knockdown of *sox2*/*3*/*19a*/*19b*, whereas loss of the transactivation domain led to loss-of-function of Sox3 in zebrafish embryos (Okuda et al., 2010). In the present study, we observed that the truncated Sox3^f7^ and Sox3^f40^ without the transactivation domain probably have no dominant negative roles to occupy competitively DNA binding sites for other Sox family proteins, owe to transcript destabilization and degradation in *sox3*^*f7*^ and *sox3*^*f40*^ mutants. A similar knockout strategy was also used in *irf6* (Li et al., 2017) and *hemogen* (Peters et al., 2018) knockout zebrafish, which showed transcripts destabilization and degradation of truncated Irf6 and Hemogen by NMD, and significantly decreased protein production. Therefore, if a few truncated transcripts were translated, the proteins might have been rapidly degraded (Chen et al., 2016; Facchinello et al., 2017). However, it cannot exclude a weak dominant-negative effect on other target genes, as Sox18 missing part of the transactivation domain may act in a dominant-negative manner (Pennisi et al., 2000a; Pennisi et al., 2000b).

Importantly, we revealed that Sox3 acts as an apoptosis suppressor in ovary development. Transcriptome analysis showed that the apoptosis signaling pathway was significantly up-regulated in *sox3*^−/−^ ovaries in comparison with wild type ovaries. Specifically, Cleaved-caspase3 was up-regulated in *sox3* knockout ovaries compared to wild type ovaries. Notably, obvious apoptosis signals were detected in both theca cells and granulosa cells of stages III and IV follicles of *sox3*^−/−^ ovaries. Thus, Sox3 can suppress apoptosis of somatic theca cells and granulosa cells in ovary. Because of the apoptosis, reduced nutrients from the somatic cells for oocyte development lead to follicle development retardation, thus fecundity decrease. Apoptosis was also up-regulated in early stage of embryo in *sox3* knockdown zebrafish (Dee et al., 2008) and overexpression of Sox3 inhibited apoptosis in epithelial ovarian cancer cell (Yan et al., 2016). In women, a high percentage of apoptosis in granulosa cells resulted in decreased ovarian fecundity (Sadraie et al., 2000) and pregnancy rate (Sifer et al., 2002). Hence, up-regulation of apoptosis of somatic cells can affect ovary function. In addition, balance between pro-apoptosis and anti-apoptosis is important for pathological and physiological processes. As a suppressor, Sox3 can balance the apoptotic level in ovary development, thus ensure normal fecundity in females.

**Figure 7:**
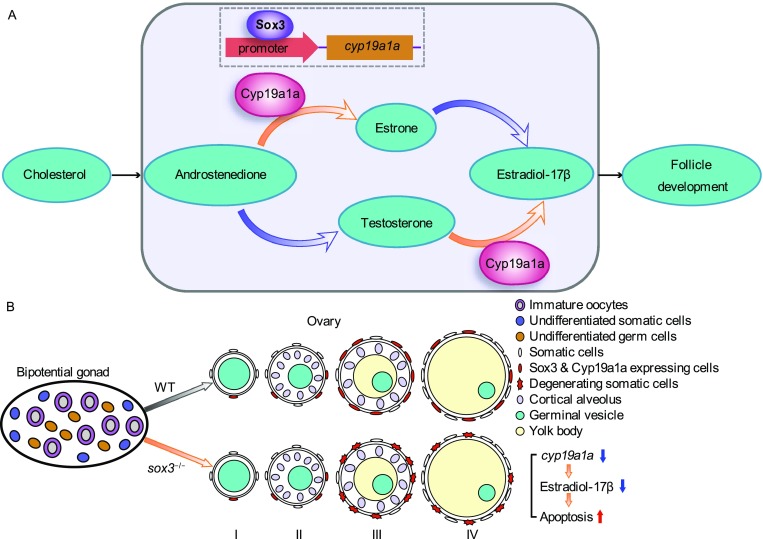
**A work model for Sox3-Cyp19a1a in ovary development in zebrafish**. (A) The pathway of 17β-E2 synthesis in ovary. Sox3 binds to the promoter of *cyp19a1a* and up-regulates the cyp19a1a expression, which promotes 17β-E2 synthesis via the two pathways, one is that androstenedione is converted to estrone and another is that testosterone is converted to 17β-E2. (B) Wild-type juvenile gonads are bipotential between 22–28 dpf post fertilization. Cyp19a1a promotes ovary development by up-regulation of 17β-E2 synthesis in wild type. In the absence of Sox3, the expression level of *cyp19a1a* is down-regulated, which results in a decrease of 17β-E2, thus leads to somatic cell apoptosis in follicles, mostly at early-vitellogenic stage (III) and late-vitellogenic stage (IV), and a reduced fecundity

Sox3 functions as a regulator of Cyp19a1a expression, via 17β-E2 linking apoptosis suppression, which is implicated in improving female fecundity. Sox3 can bind to the promoter of *cyp19a1a* and up-regulate Cyp19a1a expression in zebrafish ovary. The similar Sox3 binding site at intron 1 and activation of ovary-type *cyp19a* gene were also observed in frog (*Rana rugosa*) (Oshima et al., 2009). As a cytochrome P450 aromatase, Cyp19a1a can promote estrogen synthesis via two pathways, conversion of androstenedione to estrone, and conversion of testosterone to 17β-E2 (Nagahama, 1997). Knockout of *cyp19a1a* led to all-male offspring due to failed ovarian differentiation and the phenotype can be rescued by 17β-E2 treatment, indicating that Cyp19a1a-17β-E2 were necessary for female follicle development (Dranow et al., 2016; Lau et al., 2016; Yin et al., 2017). Thus, we determined Sox3 as the upstream regulator of Cyp19a1a-17β-E2 in ovary function. Juvenile gonads of zebrafish are bipotential between 22–28 dpf post fertilization. Cyp19a1a promotes ovary development by up-regulation of 17β-E2 synthesis in wild type. In the absence of *sox3*, the expression level of *cyp19a1a* is down-regulated, which results in a decrease of 17β-E2, thus leads to somatic cell apoptosis in follicles, mostly at early-vitellogenic stage (III) and late-vitellogenic stage (IV), and a reduced fecundity (Fig. 7A and 7B). 17β-E2 can inhibit apoptosis of granulosa cells and theca cells, probably by Fas ligand (FasL)-induced pathway (Quirk et al., 2006; Regan et al., 2018). In addition, 17β-E2 can also promote DNA synthesis in ovarian germ cells and act direct on oogonial proliferation (Miura et al., 2007). It seems that 17β-E2 has distinct roles in different cell types in ovary development. Aromatase inhibitor (fadrozole) can also cause sex-reversal from genetic female larvae to phenotypic males in Japanese flounder (Kitano et al., 2000), Nile tilapia (Kobayashi et al., 2003) and zebrafish (Uchida et al., 2004), and exposure to 17β-E2 during embryo-larvae-juvenile-life stage led to an increase proportion of females in zebrafish (Brion et al., 2004). These data support that 17β-E2 can promote ovary differentiation. In addition, FTZ-F1/SF-1 in medaka (*Oryzias latipes*) (Watanabe et al., 1999) and bovine (Michael et al., 1995), and Foxl2 in Nile tilapia (*Oreochromis niloticus*) (Wang et al., 2007a) and goat (Pannetier et al., 2006) can also up-regulate the transcription of ovary-type *cyp19a1*, whereas Dmrt1 can suppress *cyp19a1a* expression in Nile tilapia (Wang et al., 2010). Further, ovary- or brain-specific transcription factors of *cyp19a1a*/*cyp19a1b* genes remain to be identified.

## Materials and methods

### Zebrafish maintenance

Wild-type AB strain zebrafish (*Danio rerio*) were purchased from Institute of Hydrobiology of Chinese Academy of Sciences (Wuhan, China). Zebrafish strains were maintained and raised in recirculation systems at 28.5 °C under a cycle of 14 h: 10 h light/dark. All animal experiments and methods were performed in accordance with the relevant approved guidelines and regulations, as well as under the approval of the Ethics Committee of Wuhan University.

### Generation of *sox3* mutant lines and genotyping

The pGH-T7-zCas9 plasmid was linearized by X*ba*I and used as a template for *in vitro* transcription using the mMessage mMachine T7 Ultra Kit (Ambion AM1345, Austin, TX, USA). *Sox3* guide RNA (gRNA) was synthesized using a protocol according to previously described methods (Liu et al., 2014a). The oligonucleotides were synthesized by TsingKe Biotechnology (Wuhan, China) (Oligo-F: 5′ TGTAATACGACTCACTATAGGTGTCGGTGGGCCAGCGGAGTTTTAGAGCTAGAAATAGC 3′; Oligo-R: 5′ AGCACCGACTCGGTGCCACT 3′). T7 promoter sequence was added to the 5′- upstream of the oligo-F. PCR amplification using the primers and gRNA scaffold plasmid was performed to generate template for the *sox3* gRNA synthesis. The gRNA was *in vitro* transcribed using the T7 RNA Polymerase Systems (Thermo Fisher Scientific, Grand Island, NY, USA). The transcribed Cas9 mRNA and gRNA were further purified by RNeasy Mini Kit (Qiagen 74104, Germany). Embryos were collected by pair mating, maintained in Hank’s medium. Cas9 mRNA (300 ng/μL) and gRNA (20 ng/μL) were mixed and microinjected into wild-type embryos at one-cell stage using a microinjector (PV820, WPI, USA). Injected embryos were collected after 48 h for isolation of genomic DNA using NaOH-based extraction method. The target region was amplified by PCR and indel mutations were verified by sequencing. To identify germline-transmitted mutations, the injected founder embryos (F_0_) were raised to adulthood and then crossed with wild-types to generate heterozygous embryos (F_1_). Genotyping was performed by PCR amplification of target sites using caudal fin DNA and sequencing. The same indel mutants of F_1_ were intercrossed to generate homozygous embryos (F_2_). Two independent *sox3* mutant lines were established.

### Antibodies

Primary antibodies: Anti-Sox3 (GeneTex, GTX132494) was purchased from GeneTex, Irvine, California, USA. Anti-caspase3 (H-277) was from ZSGB-BIO, Beijing, China. Anti-Gapdh (glyceraldehyde-3-phosphate dehydrogenase, CW0100) was purchased from CWBIO, Beijing, China. Anti-Cyp19a1 (A12684) was from ABclonal, Wuhan, China.

Secondary antibodies: goat anti-rabbit IgG (H + L), horseradish peroxidase (HRP) conjugated antibody (31460) and goat anti-mouse IgG (H + L), HRP conjugated antibody (31430) were purchased from Invitrogen, Carlsbad, USA. FITC-conjugated immunopure goat anti-rabbit IgG (H + L) (ZF-0311) was from Feiyi Technology, Wuhan, China.

### Plasmid constructs

Full-length *sox3* (NM_001001811.2) was cloned into pcDNA3.0 using E*coR*I and X*ho*I to generate pCMV-Sox3. Five deletion fragments of the zebrafish *cyp19a1a* promoter were amplified from zebrafish genomic DNA, which were double-digested with S*ac*I and B*gl*II and cloned into the pGL3-basic vector (E1751, Promega, Madison, WI, USA). The primers and PCR conditions are described in Table S1. Site-directed mutagenesis for the two Sox3 binding sites (b and c) were performed using the primers described in Table S1. *cyp19a1a* (b^mut^) was used as the template for constructing both *cyp19a1a* (b^mut^) and *cyp19a1a* (c^mut^) mutants. All constructs were sequenced.

### RNA isolation and quantitative real-time PCR

Total RNAs of zebrafish tissues were isolated using the TRIzol Reagent (15596-026, Thermo Fisher) and then were treated with RNase-free DNase (M610A, Promega, Madison, WI, USA). The total RNAs were reverse-transcribed to complementary DNAs (cDNAs) using MMLV (M1701, Promega). SYBR Green qPCR Mix (D01010, GeneCopoeia, Rockville, MD, USA) was used for quantitative real-time PCR amplification in a StepOne real-time PCR system (Applied Biosystems, USA). The primers and PCR conditions are listed in Table S1.

### Immunofluorescence analysis

Immunofluorescence analysis was performed according to our previous study (Hou et al., 2012). Zebrafish ovaries were embedded into OCT medium (4583, Sakura Tissue-Tek, Torrance, CA, USA) and frozen at −20 °C, and then cut into a series of 6~7 μm sections using a cryostat (CM1850, Leica, Bensheim, Germany). The sections were fixed with 4% paraformaldehyde (PFA) for 20 min at room temperature and permeabilized with 1% Triton X-100 (9002-93-1, Sigma-Aldrich, USA) in PBS for 10 min and then blocked in 5% bovine serum albumin (BSA)/PBS for 30 min at room temperature. The sections were incubated with anti-Sox3 or anti-Cyp19a1 polyclonal antibody in 5% BSA/PBS overnight at 4 °C. After washing 3 times with PBS, the sections were incubated with FITC-conjugated immunopure goat anti-rabbit IgG for 1 h at room temperature. The nuclei were stained by Hoechst. Images were captured by a confocal fluorescence microscope (FV1000, Olympus, Tokyo, Japan).

### Western blot analysis

Western blots were performed according to our previous protocols (Yuan et al., 2015). Protein extracts from zebrafish ovaries were separated in 12% SDS-polyacrylamide gels and then transferred onto 0.45 μm PVDF membranes (NK0414, Roche Diagnostics, Indianapolis, IN, USA). The membranes were then blocked with 5% non-fat dried milk in TBST (20 mmol/L Tris-HCl, pH 7.5, 150 mmol/L NaCl, 0.1% Tween-20) for 1 h at room temperature. The primary antibodies were incubated with the membranes overnight at 4 °C. The membranes were washed in TBST 4–5 times, incubated with the HRP-conjugated secondary antibody for 1 h at room temperature and then washed in TBST 4–5 times. A super signal chemiluminescent substrate system (K-12045-D50, advansta, Menlo Park, USA) was used to detect the signals.

### Cell culture, transfection and dual-luciferase reporter assays

HEK293T and CHO cells were cultured in high glucose Dulbecco’s modified Eagle’s medium (DMEM) (SH30022.01B, HyClone, Logan, USA) with 10% fetal bovine serum (FBS) (P30-330250, PAN-Biotech, Aidenbach, Germany) in 12/48-well plates and Lipofectamine^TM^ 2000 (11668027, Invitrogen) was used for transfection according to the routine protocol. For luciferase assays, cells per well was transfected with 0.4 μg recombinant constructs and 10 ng pRL-TK (E2241, Promega). Then luciferase activities were measured by a dual-luciferase reporter assay system (Promega, Madison, WI, USA) and a Modulus Single Tube Multimode Reader (Turner Biosystems, Sunnyvale, CA, USA) according to the manufacturer’s protocol. The experiments were repeated at least 3 times, and the results were expressed as the means ± SD.

### Chromatin immunoprecipitation (ChIP)

Chromatin immunoprecipitation was performed according to our previous study (Fu et al., 2015). Zebrafish ovaries were cut into small pieces in PBS and a final concentration of 1% formaldehyde-PBS was used for crosslinking for 20 min at room temperature. Glycine was added to terminate crosslinking in a final concentration of 0.125 mol/L. Then the supernatant chromatin was immunoprecipitated with anti-Sox3, no antibody (beads only) or preimmune IgG together with protein G PLUS-agarose (Sc-2002, Santa Cruz, USA). The DNA isolated from the immunoprecipitated complex was amplified by PCR using primers flanking the Sox3 binding region (−493 to −112 bp) and control region (7,708 to 7,917 bp) (Table S1). The PCR products were cloned into T-easy vector (A137A, Promega, Madison, USA) and sequenced.

### Histological and TUNEL assays

Zebrafish ovaries were embedded into OCT medium (4583, Sakura Tissue-Tek) and frozen at −20 °C, and then cut into a series of 6–7 μm sections with a cryostat (CM1850, Leica). The sections were stained by haematoxylin and eosin (H.E.), and images were captured using a Leica microsystems (Leica). For TUNEL (terminal deoxynucleotidyl transferase-mediated dUTP-biotin nick end labeling) assays, TUNEL Apoptosis Assay Kit-FITC (AT005, Qihaifutai, Shanghai, China) was used according to the manufacturer’s protocol. The sections were fixed with 4% paraformaldehyde for 20 min at room temperature and permeabilized with 20 μg/mL proteinase K (P2308, Sigma-Aldrich) for 10 min at room temperature, and then the sections were incubated with the TdT mix containing FITC-dUTP for 1 h at 37 °C. The nuclei were stained by propidium iodide (PI) for 2 min. Images were captured by a confocal fluorescence microscope (FV1000, Olympus).

### Flow cytometry

For apoptosis assays, Annexin V-FITC/PI Apoptosis Kit (WLA001, Wanleibio, Shenyang, China) was used according to the manufacturer’s protocol. CHO cells were treated with 17β-E2 (500 ng/mL, E2758, Sigma-Aldrich) or DMEM (control) for 36 h, and then treated with etoposide (3 μg/mL) for 12 h and assayed by flow cytometry (CyAn ADP, Beckman Coulter, Brea, USA). The data were analyzed according to Summit 4.3 Software (Beckman Coulter).

### Enzyme-linked immunosorbent assay (ELISA)

Zebrafish ovaries and testes were cut into small pieces in cold PBS and sonication was performed using a sonicator (Sonics & Materials Inc., Newtown, USA). The homogenates were centrifuged at 3000 ×*g* for 15 min and the supernatant was used to test 17β-estradiol according to the manufacturer’s protocol (17β-E2 ELISA Kit, ml0386133, MLBIO, Shanghai, China). Concentration of 17β-E2 was determined based on standard curve of 17β-E2 (0, 87.5, 175, 350, 700, 1,400 pg/mL) at OD_450_. The optical density (OD) was read at 450 nm using a microplate reader (SpectraMax M2, Molecular Devices, USA). Data (means ± SEM) was analyzed by T-test.

### Transcriptome sequencing and analysis

Total RNAs of ovaries were extracted from each genotype (*sox3*^+/+^ or *sox3*^−/−^) of three adult individuals. The mRNA was enriched using Oligo (dT) and then broken up for cDNA library construct according to the manufacturer’s protocol. Then the cDNA library was sequenced using BGISEQ-500 platform. To determine gene expression levels, RNA-seq clean reads from *sox3*^+/+^ or *sox3*^−/−^ ovaries were mapped to the reference genome by HISAT (Kim et al., 2015) and mapped to the reference genes by Bowtie2 (Langmead et al., 2009). Fragments per kilo bases per million fragments (FPKM) values were calculated for each gene by RSEM (Li and Dewey, 2011). Differentially expressed genes (DEGs) were defined with fold change (FC) > 1.4 and false discovery rate (FDR) < 0.05. The Gene Ontology (GO) of the DEGs was analyzed by the WEGO online tool (Ye et al., 2006). For the Kyoto Encyclopedia of Genes and Genomes (KEGG) analysis, the DEGs were implemented to map KEGG public database according to a previous study (Kanehisa et al., 2008).

### Statistical analysis

All data were presented as means ± standard error of mean from at least three independent experiments. Statistical comparisons were made using Student’s* t*-test when comparing two groups. One-way ANOVA was performed for comparisons with more than two groups. Statistics analysis was performed using GraphPad Prism 6 software package (GraphPad Software, La Jolla, USA). In all analysis, *P* < 0.05 was considered to be statistically significant.

### Data availability

Strains are available upon request. Transcriptome data were deposited in the GEO database (accession no. GSE115806). Supplemental information including materials and methods, four figures and four tables can be found in supplementary file. All primers used in this study are listed in Table S1.

## Electronic supplementary material

Below is the link to the electronic supplementary material.

Supplementary material 1 (PDF 640 kb)
