# Supplementary material for: Loss-of-function of sox3 causes follicle development retardation and reduces fecundity in zebrafish
Source: Protein Cell. 2018 Dec 26;10(5):347–64. doi: 10.1007/s13238-018-0603-y (PMC6468042; doi:10.1007/s13238-018-0603-y)
Supplement: Supplementary file 1 — Supplementary material 1 (PDF 640 kb) [file 13238_2018_603_MOESM1_ESM.pdf]

Loss-of-function of *sox3* causes follicle development retardation and reduces fecundity in zebrafish

Running title: Follicle development retardation in *sox3* KO zebrafish

Qiang Hong[1], Cong Li[1], Ruhong Ying, Heming Lin, Jingqiu Li, Yu Zhao, Hanhua Cheng*, Rongjia Zhou*

Hubei Key Laboratory of Cell Homeostasis, College of Life Sciences, Wuhan University, Wuhan 430072, P. R. China

*Corresponding authors: Professors Rongjia Zhou and Hanhua Cheng, College of Life Sciences, Wuhan University, Wuhan 430072, P. R. China, Fax: 0086-27-68756253, E-mail: rjzhou@whu.edu.cn, hhcheng@whu.edu.cn

1, Co-first authors: Qiang Hong and Cong Li

SUPPLEMENTAL MATERIALS AND METHODS

p5xGal4-CMV-luc (Addgene plasmid # 46322) (Kowalska et al., 2012) was purchased from Addgene (Cambridge, MA, USA). Full-length Sox3, Sox3^{f7}, Sox3^{f40}, Sox3-N and Sox3-C were cloned into pGBKT7 (Clontech, USA) using E*coR*I and B*am*HI to generate pGal4-Sox3, pGal4-Sox3^{f7}, pGal4-Sox3^{f40}, pGal4-Sox3-N, pGal4-Sox3-C, and then were cloned into pCMV-Tag2B (Stratagene, USA) using B*am*HI and S*al*I to generate Gal4-Sox3, Gal4-Sox3^{f7}, Gal4-Sox3^{f40}, Gal4-Sox3-N, Gal4-Sox3-C and Gal4. The primers and PCR conditions are described in Table S1. All constructs were sequenced. For luciferase assays, cells per well was transfected with 0.4 μg recombinant constructs and 10 ng pRL-TK (E2241, Promega). Then luciferase activities were measured by a dual-luciferase reporter assay system (Promega, Madison, WI, USA) and a Modulus Single Tube Multimode Reader (Turner Biosystems, Sunnyvale, CA, USA) according to the manufacturer’s protocol. The experiments were repeated at least 3 times, and the results were expressed as the means ± SD.

REFERENCE

Kowalska E, Ripperger JA, Muheim C, Maier B, Kurihara Y, Fox AH, Kramer A, Brown SA (2012) Distinct roles of DBHS family members in the circadian transcriptional feedback loop. Mol Cell Biol 32: 4585-4594

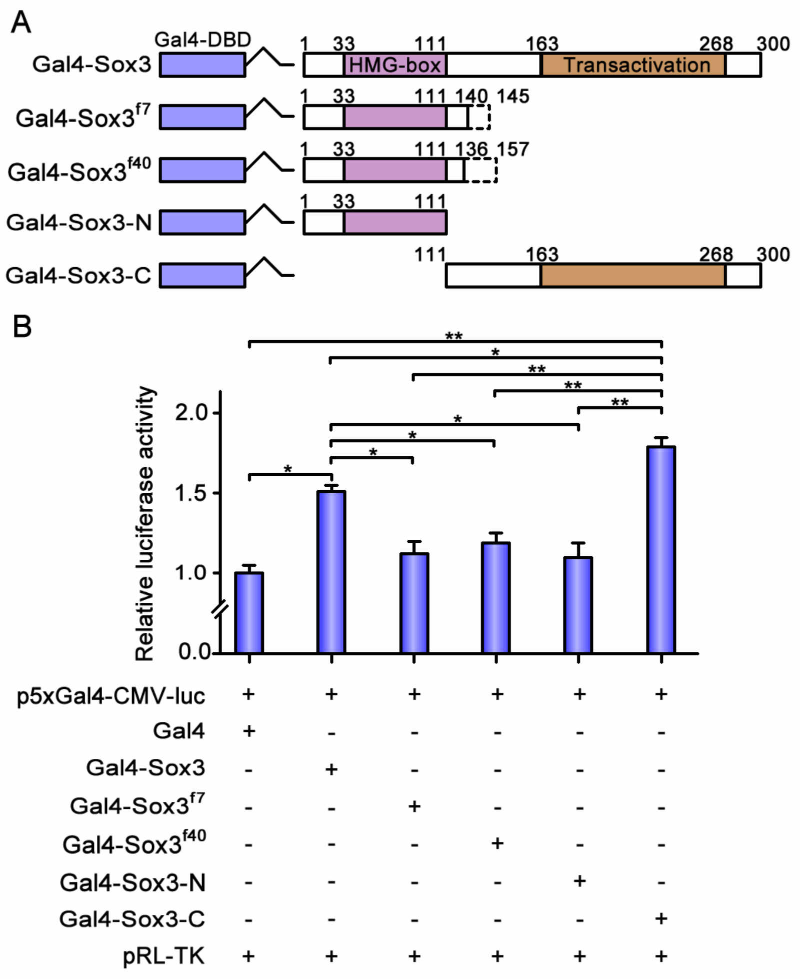

Figure S1. Sox3 domain analysis.

(A) A schematic diagram of wild type and deletion constructs of zebrafish Sox3. The conserved domains (HMG-box and transactivation domain) are indicated in wild type Sox3. The DNA binding domain (amino acids 1–147) of Gal4 was fused to amino acids 1–300 of Sox3 in Gal4-Sox3, to aa 1–145 of Sox3^{f7} in Gal4-Sox3^{f7}, to aa 1–157 of Sox3^{f40} in Gal4-Sox3^{f40}, to aa 1–111 of Sox3 in Gal4-Sox3-N, and to aa 111–300 of Sox3 in Gal4-Sox3-C, respectively. (B) HEK293T cells were transfected with 0.2 μg p5xGal4-CMV-luc (Addgene plasmid # 46322) and 0.2 μg Sox3 expression plasmid (Gal4-Sox3) or its several constructs (Gal4, Gal4-Sox3^{f7}, Gal4-Sox3^{f40}, Gal4-Sox3-N and Gal4-Sox3-C), together with 10 ng pRL-TK, as indicated. One-way ANOVA was performed. *, $p < 0.05$; **, $p < 0.01$.

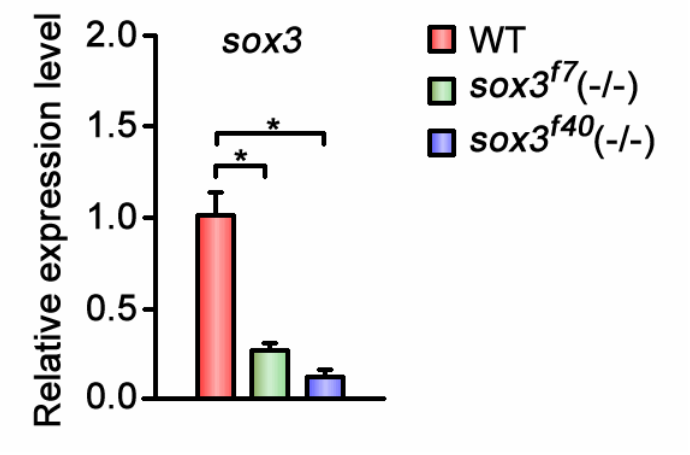

Figure S2. The expression of *sox3* in *sox3* knockout ovaries and wild type ovaries.

Quantitative real-time PCR was used to analyze the expression of *sox3* gene in *sox3* knockout ovaries and wild type ovaries. The transcript levels were related to *β-actin* expression. Relative level, $2^{-\Delta\Delta Ct}$. T-test was performed. *, $p < 0.05$.

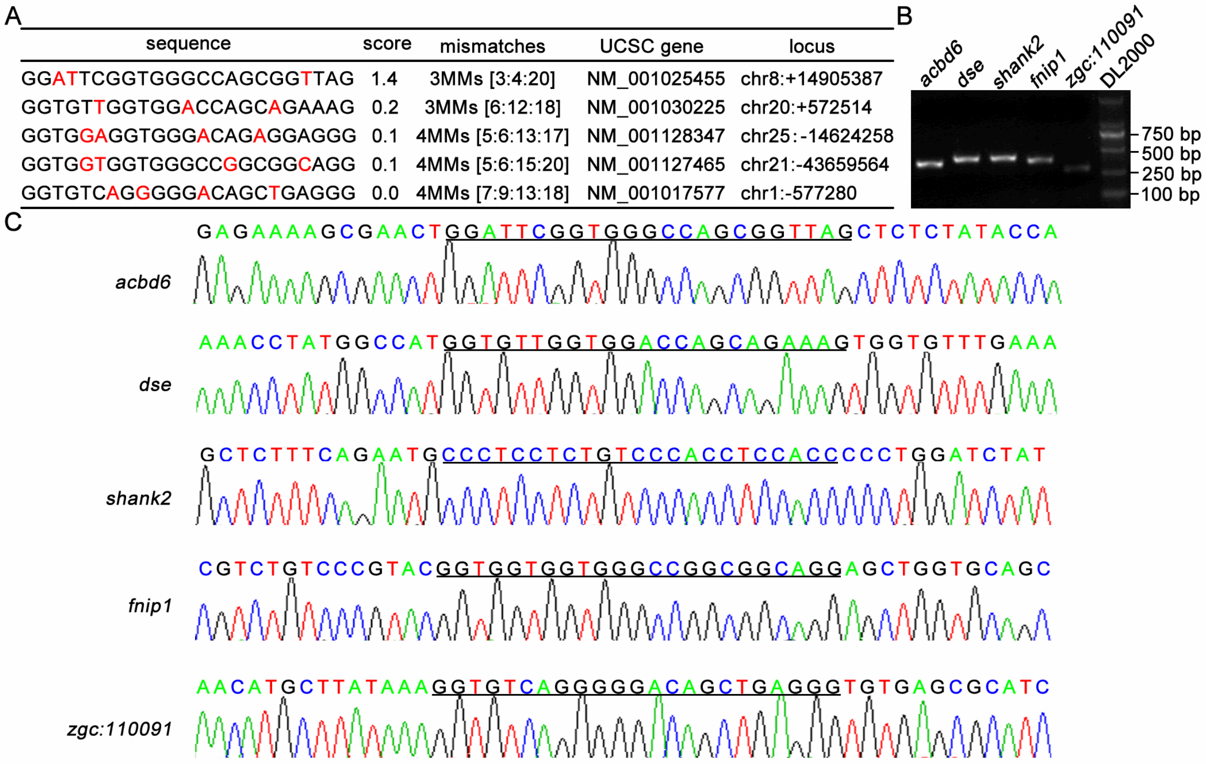

Figure S3. Off-target analysis.

(A) The predicted off-target sites was aligned with zebrafish genome. (B) PCR amplification of predicted off-target sequences. The caudal fins of homozygotes were collected for genomic DNA extraction and PCR amplification was performed. (C) The PCR products were sequenced. The off-target sites were underlined in black.

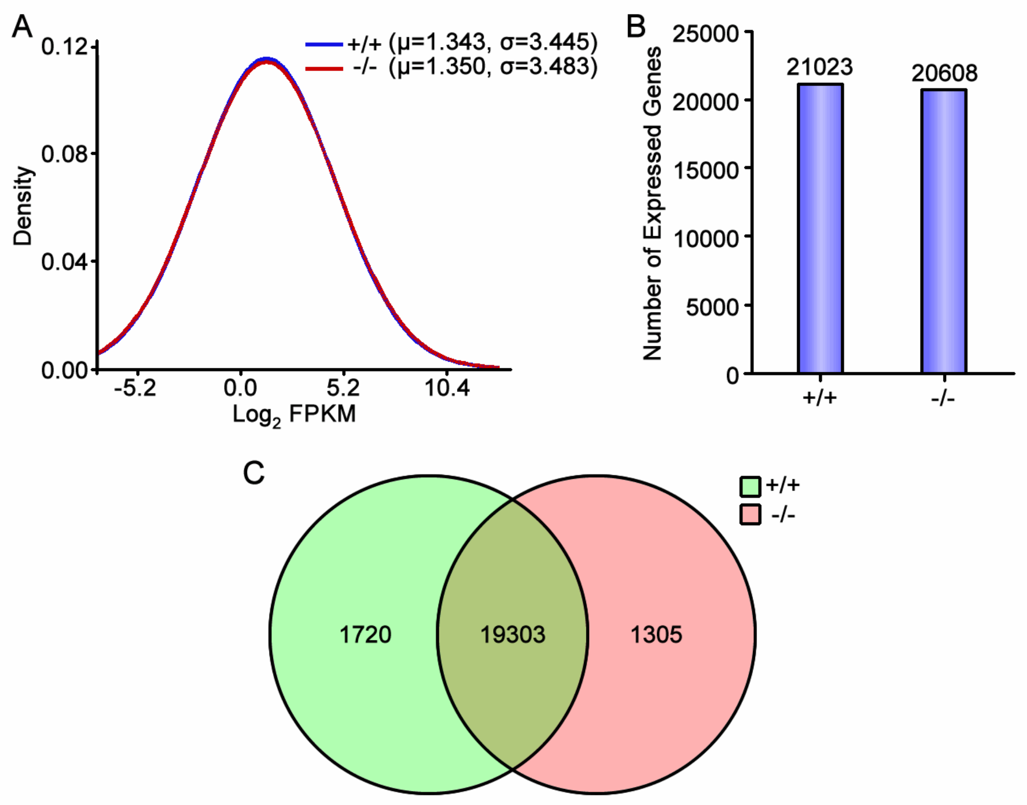

Figure S4. Transcriptome data.

Numbers of expressed genes in the ovaries between *sox3*$^{+/+}$ and *sox3*$^{-/-}$ based on RNA-seq data. (A) The distribution of gene density according to gene expression levels in both *sox3*$^{+/+}$ and *sox3*$^{-/-}$ ovaries. (B) Numbers of expressed genes in both *sox3*$^{+/+}$ and *sox3*$^{-/-}$ ovaries from RNA-seq data. (C) Venn chart indicated the co-expressed and ovary-specific expressed genes between *sox3*$^{+/+}$ and *sox3*$^{-/-}$ ovaries.

Table S1. Primer sequences and PCR conditions

Genes/fragments	GenBank access No.	Primer sequence (5’-3’)	Tm (℃)
sox3 (genotyping)	NM_001001811.2	F: GCGCCTCGGTGCTGACTG<br>R: TAGGCCAGCTGGTCCTGCAT	60
sox3 (CDS)	NM_001001811.2	F: CGGAATTCATGTATAACATGATGGAAACCG<br>R: CGCTCGAGAATGTGGGTTAGGGGTAGCGT	58
β-actin	NM_131031.1	F: GGGAGTGATGGTTGGCATGG<br>R: AGGAAGGAAGGCTGGAAGAG	60
acbd6	NM_001025455.1	F: TTGATTCACTGAAGCGAGTA<br>R: GCAGGAAAATGAATCATGGC	58
dse	NM_001030225.1	F: AGGCAGTAGTGAGAAAGCAG<br>R: GTGTTAAGCACCCGAGAAAG	58
shank2	NM_001128347.1	F: TGACTCGGGCATTGAGGTAG<br>R: GGCTTTGGTGCTTTGTCCTT	58
fnip1	NM_001127465.2	F: GGGTACAGGGAGCTACTCTACT<br>R: TGGTGTTCGTCTGGGTTTGC	58
zgc:110091	NM_001017577.1	F: GCTCAGGTGGGCGTGTCTGT<br>R: TCGGGCTCATCTGTGACTGC	58
cyp19a1a	NM_131154.3	F: TGGGTCCTGTTGTCTCCTAC<br>R: AGTTTACTTCCAAAGCGTGA	58
cyp19a1a (P1)	NM_131154.3	F: ATAGAGCTCTGGCATCATGGACAAAGAACA<br>R: ATAAGATCTGCAAGTCTAAAGCCTCTGAACT	60
cyp19a1a (P2)	NM_131154.3	F: ATAGAGCTCAGGTGCATCAAAATAAGGACAC<br>R: ATAAGATCTGCAAGTCTAAAGCCTCTGAACT	60
cyp19a1a (P3)	NM_131154.3	F: ATAGAGCTCAGGCCTGATGTTTTCTCATTT<br>R: ATAAGATCTGCAAGTCTAAAGCCTCTGAACT	60
cyp19a1a (P4)	NM_131154.3	F: ATAGAGCTCTGAAGTCCTGATGAAAACCCA<br>R: ATAAGATCTGCAAGTCTAAAGCCTCTGAACT	60
cyp19a1a (P5)	NM_131154.3	F: ATAGAGCTCTATCCTGATTGAGTCCCATGC<br>R: ATAAGATCTGCAAGTCTAAAGCCTCTGAACT	60
cyp19a1a (b^{mut})	NM_131154.3	F: AGCGTTTTGTAGGCCTGATGTTTTC<br>R: GCGGCGTGTCCTTATTTTGATGCAC	58
cyp19a1a (c^{mut})	NM_131154.3	F: AGCGTTAGGCCTGATGTTTTCTCAT<br>R: CGCTCAAAAGTGTCCTTATTTTGAT	58
cyp19a1a (b/c^{mut})	NM_131154.3	F: GTTAGGCCTGATGTTTTCTCATTTGAC<br>R: GCACGCTGCGGCGTGTCCTTAT	58
cyp19a1a (ChIP-P1)	NM_131154.3	F: CCAGAAATGTATATAAAGGGTACATAT<br>R: AGTCATCTCTGGGTTTTCATCAGG	57
cyp19a1a (ChIP-P2)	NM_131154.3	F: CACAAACTCTCACCTGGACGA<br>R: TCCCATATAGAAACTGTGGTCTTA	57
casp3a	NM_131877.3	F: GCCAAGCCTCAATCCCAT<br>R: GCCGAAAAACACCCCCTC	58

tspo	NM_001006032.2	F: AGGTATAATCACACGGCGGGA<br>R: CCACTGTGCCACTCATCAACA	58
pmaip1	NM_001045474.3	F: CAAACCGCTGTAGTAGAGTGC<br>R: ATCGCTTCCCCTCCATTTGTA	58
capn12	NM_001083063.2	F: AATCACCAGCAATGCCGTCT<br>R: CGTTGCTGGTGCGAGAGTAG	58
boka	NM_001003612.2	F: AGGTGTTTGATCGCTCTCCCA<br>R: CTCATCACCCAACCACAGCAG	58
pgal4-sox3	NM_001001811.2	F: CGGAATTCTATAACATGATGGAAACCGAGA<br>R: CGGGATCCGTCAAATGTGGGTTAGGGGTAG	58
pgal4-sox3^{f7}	NM_001001811.2	F: CGGAATTCTATAACATGATGGAAACCGAGA<br>R: CGGGATCCGTCATGTGCGTGTAGTCCAT	58
pgal4-sox3^{f40}	NM_001001811.2	F: CGGAATTCTATAACATGATGGAAACCGAGA<br>R: CGGGATCCGTCATACTGGGATGTTGAGGG	58
pgal4-sox3-N	NM_001001811.2	F: CGGAATTCTATAACATGATGGAAACCGAGA<br>R: CGGGATCCGGGTCTTGGTCTTCCTGCG	58
pgal4-sox3-C	NM_001001811.2	F: CGGAATTCCTGCTGAAGAAAGACAAGTATTCT<br>R: CGGGATCCGTCAAATGTGGGTTAGGGGTAG	58
gal4-sox3	NM_001001811.2	F: CGGGATCCATGAAGCTACTGTCTTCTAT<br>R: CGGTCGACTCAAATGTGGGTTAGGGGTAG	60
gal4-sox3^{f7}	NM_001001811.2	F: CGGGATCCATGAAGCTACTGTCTTCTAT<br>R: CGGTCGACTCATGTGCGTGTAGTCCAT	60
gal4-sox3^{f40}	NM_001001811.2	F: CGGGATCCATGAAGCTACTGTCTTCTAT<br>R: CGGTCGACTCATACTGGGATGTTGAGGG	60
gal4-sox3-N	NM_001001811.2	F: CGGGATCCATGAAGCTACTGTCTTCTAT<br>R: CGGTCGACGGTCTTGGTCTTCCTGCG	60
gal4-sox3-C	NM_001001811.2	F: CGGGATCCATGAAGCTACTGTCTTCTAT<br>R: CGGTCGACTCAAATGTGGGTTAGGGGTAG	60
gal4		F: CGGGATCCATGAAGCTACTGTCTTCTAT<br>R: CGGTCGACGAATTCGGCCTCCATGGCCA	60
sox3 (RT-PCR)	NM_001001811.2	F: ACCGAGATTAAAAGCCCCAT<br>R: TTGCTGATCTCCGAGTTGTG	59

The restriction sites were underlined.

Table S2. Summary of transcriptome data from *sox3*$^{+/+}$ and *sox3*$^{-/-}$ ovaries

Samples	Sequencing Strategy	Raw Data Size (bp)	Raw Reads Number	Clean Data Size (bp)	Clean Reads Number	Clean Data Ratio (%)
KO-ovary	SE50	1175061850	23501237	1173123100	23462462	99.83
WT-ovary	SE50	1178747350	23574947	1176463600	23529272	99.80

Clean data ratio (%) = Clean reads number/raw reads number

Table S3. Alignment statistics of clean reads to reference genome

Samples	Total Reads	Total Mapped Reads (%)	Unique Match (%)	Multi-position Match (%)	Total Unmapped Reads (%)
KO-ovary	23462462	91.36	69.74	21.62	8.64
WT-ovary	23529272	91.82	70.99	20.83	8.19

Table S4. Alignment statistics of clean reads to reference genes

Samples	Total Reads	Total Mapped Reads (%)	Unique Match (%)	Multi-position Match (%)	Total Unmapped Reads (%)
KO-ovary	23462462	86.32	73.00	13.33	13.68
WT-ovary	23529272	86.15	73.19	12.96	13.85
